# Effects of Nonpharmacological Interventions on Sleep Quality and Insomnia in Perimenopausal and Postmenopausal Women: A Meta-Analysis

**DOI:** 10.3390/healthcare11030327

**Published:** 2023-01-22

**Authors:** Beomman Ha, Jisoon Kim, Wi-Young So, Seonho Kim

**Affiliations:** 1 Armed Forces Medical Command, Seongnam-si 13574, Republic of Korea; 2 Department of Nursing, Woosong University, Daejeon-si 34606, Republic of Korea; 3 Sports Medicine Major, College of Humanities and Arts, Korea National University of Transportation, Chungju-si 27469, Republic of Korea; 4 Department of Nursing Science, College of Medicine, Chungbuk National University, Cheongju-si 28644, Republic of Korea

**Keywords:** sleep quality, insomnia, women, menopause, meta-analysis

## Abstract

This study aimed to analyze the effects of nonpharmacological interventions on perimenopausal and postmenopausal women with sleep problems. Eight databases (MEDLINE/PubMed, Cochrane Library, EMBASE, CINAHL, and four Korean databases) were searched, from their inception through to 30 November 2021, for randomized controlled trials (RCTs) evaluating the effects of nonpharmacological interventions versus control conditions on sleep quality and insomnia in perimenopausal and postmenopausal women. Sleep quality was assessed using the Pittsburgh Sleep Quality Index (PSQI), and the severity of insomnia was assessed using the Insomnia Severity Index (ISI). In the meta-analysis, corrected standardized mean differences (SMDs; Hedges’ g) and 95% confidence intervals (CIs) were calculated as effect measures by applying the random effects model and inverse variant method. Fifteen trials met our inclusion criteria. Nonpharmacological interventions were found to have positive effects on sleep quality, measured with the PSQI (SMD = −1.32; 95% CI = −1.78 to −0.86; *p* < 0.001), and on the severity of insomnia, measured using the ISI (SMD = −1.11; 95% CI = −1.82 to –0.41; *p* = 0.002), compared with the control groups. Among perimenopausal and postmenopausal women with sleep problems, nonpharmacological interventions improved sleep quality and reduced the severity of insomnia.

## 1. Introduction

Menopause occurs naturally in most women between the ages of 45 and 52 years old and is marked by changes in hormonal status and the cessation of the menstrual cycle [1,2,3]. During this time, women experience vasomotor, physical, and physiological problems, which reduce their quality of life [4]. Menopausal symptoms include hot flashes, night sweats, sleep disorders, sexual dysfunction, mood disorders, weight gain, and cognitive decline. Among menopausal symptoms, sleep disorders are one of the most troublesome and are reported in 40–56% of menopausal women [5,6]. Menopausal symptoms such as hot flashes and night sweats cause frequent awakenings and sleep disturbances in menopausal women [6]. Sleep disturbances affect health-related quality of life, work productivity, and healthcare utilization [7], and can have negative effects on one’s physical and psychological health and well-being over the several years of menopausal transition [8,9].

Currently, options for treating sleep disorders include pharmacological treatments and nonpharmacological interventions. Pharmacological treatments can provide short-term relief, but long-term usage can lead to various side-effects, such as drug dependency and tolerance, possible misuse, and decreased cognitive function during daytime hours [10,11,12,13]. Therefore, pharmacological treatments are mainly used for short-term treatment, whereas nonpharmacological interventions are preferred for people with chronic sleep disorders [12,14,15,16]. Nonpharmacological interventions have similar effects as pharmacological treatments and have even, at times, been reported as being more effective [17]. Previous studies have confirmed the effectiveness of nonpharmacological interventions such as cognitive behavioral therapy (CBT) (A7, A9, and A12), sleep hygiene (A13), exercise (A5 and A15), yoga (A4 and A5), meditation (A6 and A13), acupuncture (A1, A2, A8, A10, and A14), and aromatherapy (A11). The use of nonpharmacological interventions has increased in recent years [18,19].

Until recently, meta-analyses of nonpharmacological interventions for sleep disorders were dominated by studies on adult women in general, older people, and breast cancer survivors [19,20], and few studies have been conducted regarding menopausal women. Moreover, to the best of our knowledge, no studies have comprehensively analyzed various nonpharmaceutical intervention effects. Therefore, this study aimed to determine the effects of nonpharmacological interventions on sleep problems using a meta-analysis of randomized controlled trials (RCTs) in perimenopausal and postmenopausal women. Our findings contribute to the basic data necessary to develop an effective nonpharmacological intervention program for menopausal women with sleep disorders.

## 2. Methods

This meta-analysis was conducted based on the Cochrane Collaboration Handbook and Preferred Reporting Items for Systemic Review and Meta-analysis (PRISMA) statement [21]. An ethical statement was not required for this study because it is a meta-analysis based on published studies.

### 2.1. Criteria for Inclusion and Exclusion

The research question was: “Are non-drug interventions effective in improving the sleep status of menopausal women with sleep disorders?” The data for the analysis were selected according to the PICO criteria (participants, intervention, comparison, and outcomes). Studies (or study arms) were included if they met the following criteria: (1) participants included perimenopausal and postmenopausal women with sleep problems who were not cancer survivors; (2) the intervention was nonpharmacological in nature, including interventions such as CBT, sleep hygiene, exercise, yoga, meditation, acupuncture, and aromatherapy; (3) the control group was given usual care or placebo care; (4) the outcomes, such as subjective sleep quality or disturbance, were measured using validated or standardized tools such as the Pittsburgh Sleep Quality Index (PSQI) or Insomnia Severity Index (ISI); and (5) the study design was an RCT. Furthermore, we restricted our search to trials published in Korean or English between 2000 and 2021.

Nonrandomized studies, those on children and adolescents, those on cancer survivors, and those on sleep disorders caused by other causes such as obstructive sleep apnea, restless legs syndrome, and neurological diseases, were excluded. Finally, reviews, commentaries, and case reports were also excluded.

### 2.2. Search Methods

Comprehensive searches were conducted across the following electronic databases from their inception through to 30 November 2021: four English-language databases (MEDLINE/PubMed, Cochrane Library, EMBASE, and CINAHL) and four Korean-language databases (KoreaMED, Research Information Sharing Service (RISS), National Discovery for Science Library (NDSL), and Korean studies information service system (KISS)). In addition, we manually searched the bibliographies of the searched studies for further relevant studies.

The search strategy resulted in the identification of studies which included a combination of different types of participants, interventions, and outcomes. The literature search was conducted around the search terms: “perimenopausal women or postmenopausal women” and “sleep.” These were adapted for each database as necessary. The complete search strategy for PubMed was as follows: (“perimenopausal women” OR “postmenopausal women”) AND (“sleep” OR “sleep quality” OR “insomnia”).

### 2.3. Study Selection and Data Extraction

Duplicate entries from the database search were removed using a bibliographic management program (Endnote X20). Subsequently, an initial screening of the titles and abstracts was independently conducted by two authors (BH and SK) using the inclusion/exclusion criteria. The full text of the article was obtained to determine if the study met the inclusion/exclusion criteria, and their eligibility was independently determined by the reviewers. If no consensus was met on the possible inclusion/exclusion of any individual study, a final consensus decision was made by discussion with our coauthors (JK and WYS).

Data were extracted using a standardized data collection form. The extracted items included characteristics of the study (author, year, country, and study design), participants (age and sample size), interventions (type of intervention, duration and frequency of a session, and total length of intervention time), control interventions (type, frequency, length, and duration), and outcomes (outcome measures with sleep quality and insomnia). After coding independently, two researchers compared their results and, if there was a discrepancy, the original text was read together and an agreed-on coding value was used.

### 2.4. Risk of Bias in Individual Studies

The risk of bias in the included studies was assessed using the revised version of the Cochrane risk-of-bias tool for randomized trials (RoB 2.0) [22]. This tool evaluates the risk of bias according to five domains: (1) the randomization process, (2) deviation from the intended intervention, (3) missing outcome data, (4) the measurement of outcomes, and (5) the selection of the reported results. Two authors (BH and SK) independently assessed all included studies for the risk of bias, and disagreements were resolved via consensus. Each domain was rated as “low risk of bias,” “some concern,” or “high risk of bias.” The overall risk of bias for each trial was determined based on the highest risk attributed to any domain. Overall bias was considered as a “low risk of bias” if the study was classified as low risk in all domains, “some concern” if there was at least one domain rated as having some concerns, and a “high risk of bias” if there was at least one domain rated as high risk, or several domains rated as having some concerns that could affect the validity of the results.

### 2.5. Statistical Analysis

We estimated Hedges’ g, which is a corrected standardized mean difference (SMD), and its 95% confidence interval (CI) to estimate the average effect across studies. The inverse of variance was used as the weight for each effect size [23]. A random effects model was used to estimate the overall effects, assuming that the effects would be different depending on the characteristics of the participants and intervention. Statistical heterogeneity of intervention effects was measured using Cochran’s Q test and I^2^ statistics. Heterogeneity was considered substantial if the *p*-value was <0.10 in Cochran’s Q test, or the value of I^2^ statistic was >50% [24]. A moderate analysis was performed (using meta-ANOVA and meta-regression) to identify the possible reasons for interstudy heterogeneity. Finally, a visual inspection of funnel plots and Egger’s regression test [25] were used to assess publication bias. Egger’s regression test was used to determine whether the funnel plot was symmetrical (*p* < 0.05).

Statistical analysis was performed using the R software package Meta version 3.4.0 (R Foundation for Statistical Computing, Vienna, Austria) and Review Manager version 5.3 (Nordic Cochrane Center, Copenhagen, Denmark).

## 3. Results

### 3.1. Study Selection

**Figure 1:**
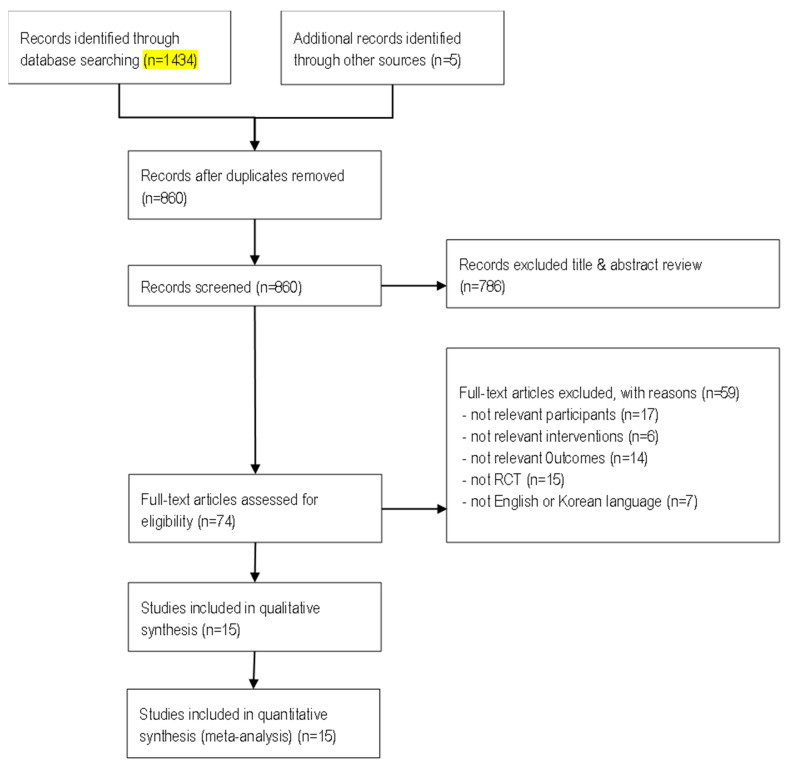
Flow diagram of study selection.

The results of the literature search and screening process are shown in Figure 1. In total, 1434 articles were retrieved by the database search. We identified five articles that met the inclusion criteria by examining the bibliographies of the selected articles. After removing duplicates, 860 articles remained. Of these, 786 articles that did not meet the inclusion criteria were excluded after examining the titles and abstracts. After 74 full-text articles were assessed for their eligibility by evaluating them in their entirety, 59 articles were excluded. Finally, we were left with 15 articles for meta-analysis.

### 3.2. Characteristics of Selected Studies

The characteristics of the 15 selected studies are summarized in Table 1. Only one article (6.7%) was published between 2006 and 2010, and three (20.0%) were published between 2011 and 2015, whereas eleven articles (73.3%) were published after 2016. This indicates that the number of studies on this subject has increased. Of the fifteen studies selected for analysis, five were conducted in Brazil, three in Iran, two in the USA, two in China, and the other three were administered in Korea, Spain, and Canada, respectively. Acupuncture was the most common intervention (n = 5), followed by CBT (n = 3), yoga (n = 2), Pilates (n = 2), walking (n = 2), meditation (n = 2), and aromatherapy (n = 1). The duration of intervention was 8 weeks for four studies, 4 weeks for three studies, 12 weeks for three studies, 16 weeks for two studies, and 3, 5, and 6 weeks for the three other studies, respectively. The PSQI was the most common method used to measure sleep quality, as it was used in eight studies (n = 8). Meanwhile, the ISI was used in one study, and both the PSQI and ISI were used in six studies. As a type of comparative intervention, nonintervention was the most common (n = 7), followed by a placebo (or sham) intervention (n = 4), usual education programs such as sleep hygiene (n = 3), and usual counseling (n = 1).

### 3.3. Quality Assessment of Selected Studies

The results of the evaluation of the risk of bias are presented in Table 1. After applying the RoB 2.0 tool, nine studies were found to have a low risk of bias in all domains, four showed a “high risk of bias or some concerns” in terms of “randomizing process,” three showed a “high risk of bias or some concerns” in terms of “measurement of outcome,” and two showed “some concerns” in terms of “selection of the report results.” Additionally, all studies had a “low risk of bias” in terms of “deviation from the intended intervention” and “missing outcome data.” Thus, the overall bias levels were low for nine studies, there was some concern for three studies, and were high for three studies.

### 3.4. Overall Effects of Nonpharmacological Intervention

**Figure 2:**
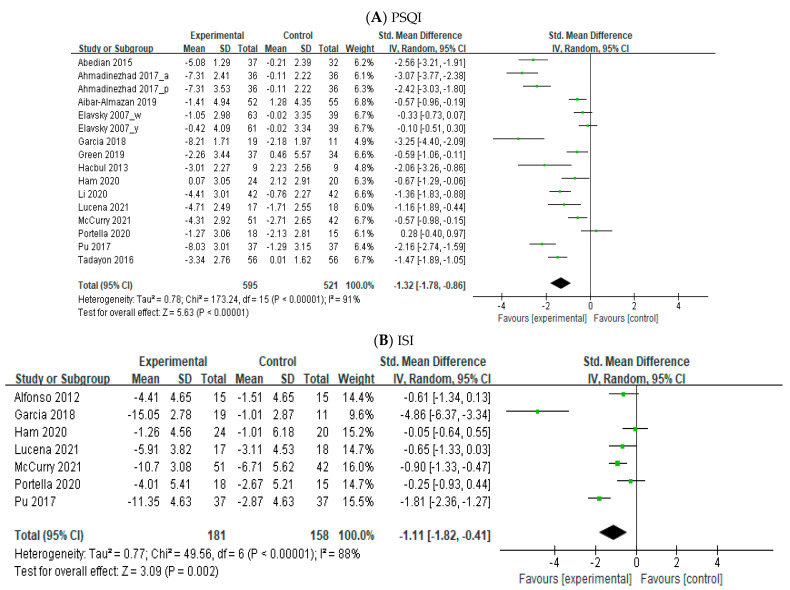
Forest plots [26,27,28,29,30,31,32,33,34,35,36,37,38,39,40].

The random effects model was applied to analyze 15 RCTs’ outcomes using different sleep outcome measurement tools (PSQI and ISI). The meta-analysis revealed the effects of nonpharmacological interventions on sleep quality and insomnia compared with the control group in perimenopausal women using the PSQI and ISI (Figure 2). Sixteen RCTs showed evidence for the positive effects of nonpharmacological interventions compared with the control group in improving sleep quality in perimenopausal women using the PSQI (SMD = −1.32; 95% CI = −1.78~-0.86; *p* < 0.001). The range of the effect size was between −3.25 (A6) and −0.57 (A12), and three RCTs were not effective. Seven RCTs contained evidence for the positive effects of nonpharmacological interventions compared with the control group in reducing the severity of insomnia in perimenopausal women using the ISI (SMD = −1.11; 95% CI = −1.82~-0.41; *p* = 0.002). The range of the effect size was between −4.86 (A6) and −0.90 (A12), and four RCTs were not effective.

### 3.5. Moderator Analysis

Significant heterogeneity existed among all studies (Q = 173.24, df = 15, *p* < 0.001, and 1^2^ = 91% using the PSQI; Q = 49.56, df = 6. *p* < 0.001; and 1^2^ = 88% using the ISI). Therefore, moderator and meta-regression analyses were conducted to explore the determinants of heterogeneity further. The studies that employed the PSQI showed that the study region and duration of intervention were significant factors in the studies, but the studies that employed the ISI indicated no significant factors in the studies (Table 2). In studies using the PSQI as an outcome measurement tool, those conducted in Asian countries showed more improvement in sleep quality than those conducted in Western countries (Hedges’ g = −1.099 vs. −0.811, *p* = 0.010). Regression analyses revealed a positive correlation between the duration of intervention (*p* = 0.010) and total length of class time (*p* = 0.019), indicating that a longer duration of intervention and greater total length of class time increased the chance of obtaining significant results.

### 3.6. Publication Bias

**Figure 3:**
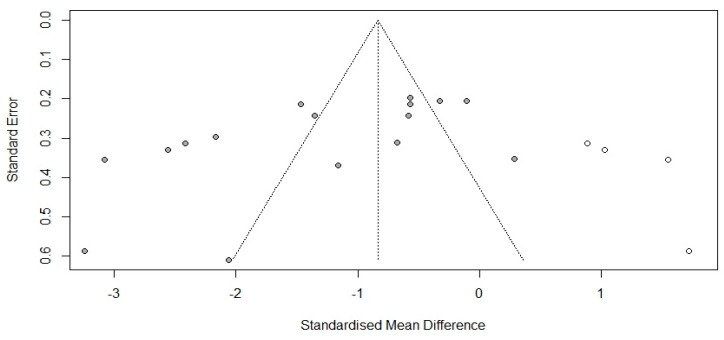
Adjusted funnel plot of Pittsburgh Sleep Quality Index.

To evaluate the potential publication bias, we used funnel plots and Egger’s regression test. In studies using the ISI, the funnel plot was symmetric, and Egger’s regression test was not significant (*p* = 0.310), indicating no publication bias. However, in studies using the PSQI, the funnel plot was asymmetric, and Egger’s regression test identified substantial asymmetry (*p* = 0.021). A sensitivity analysis using the trim-and-fill method was performed with four imputed studies, which produced a symmetrical funnel plot (Figure 3). Using the trim-and-fill method, the adjusted average effect size was calculated as -0.837, which was lower than the observed average effect size of −1.335 (95% CI = −1.47~−0.21). It can be concluded that the publication bias analysis did not appear to be at a high enough level to suggest that nonpharmacological interventions meant to address perimenopausal women’s sleep disorders were not effective.

## 4. Discussion

This study is the first meta-analysis to examine the effects of nonpharmacological interventions in improving sleep quality and reducing insomnia severity in menopausal women. We found that nonpharmacological interventions have a significant effect on improving sleep disorders in menopausal women.

Of the 15 studies included in the analysis, 11 were conducted after 2015 and accounted for 73.8% of the total, indicating that nonpharmacological intervention studies have recently been increasing. This reflects the recent trend of increasing access to nonpharmacological interventions, as there are risks associated with pharmacological interventions, including the suppression of rapid-eye-movement sleep, drug interactions, drug abuse, and side effects of long-term use such as difficulty in discontinuing use due to physical and psychological dependence [17]. Since menopausal women experience sleep disorders more frequently than other gender and age groups, it is necessary to actively conduct research using them as participants.

Bias is a systematic error meaning the deviation from the true value in the result or estimation, and bias can be a factor in underestimating or overestimating the effect of an intervention; therefore, it is important to evaluate the risk of bias in each study [41]. Using the evaluation algorithm presented in RoB 2.0, we found nine studies to have a low risk of bias, three indicated a high level of bias, and three presented slight concerns about the risk of bias. Although two studies [32,39] were evaluated as having a high risk of bias due to an insufficient description of their randomization process, we evaluated it as a problem arising from inadequate reporting of the process in the paper. As the degree of bias of the study included in the meta-analysis greatly affects the results of the meta-analysis [42], future RCTs should systematically plan and specifically describe the randomization process and the evaluator’s blind spots, thereby reducing bias, rather than just focusing on revealing the significance level of the program’s effectiveness. Furthermore, among the studies included in this meta-analysis, very few have reported the stability and side effects of the intervention. Some interventions may have negative side effects [43]; therefore, researchers need to make efforts to provide safe intervention options to expand the practical use of nonpharmacological interventions in the future.

The PSQI and ISI were used as outcome variables in this study and are widely used as primary measurement tools for sleep problems. The PSQI is a self-reported questionnaire measuring general sleep quality, and the ISI is associated with functional impairment as a tool for evaluating the severity of insomnia symptoms [44,45]. In previous studies [46,47], the effect size differed according to the outcome variables (PSQI and ISI); therefore, in this study, the outcome variables were classified and analyzed using the PSQI and ISI.

The overall effect size of nonpharmacological interventions on the quality of sleep as measured by the PSQI and the severity of insomnia as measured by the ISI in menopausal women was –1.32 (95% CI = −1.78~−0.86; *p* < 0.001) and −1.11 (95% CI = −1.82~−0.41; *p* = 0.002), respectively. In other words, nonpharmacological interventions for menopausal women with sleep disorders were found to have a positive effect on quality of sleep and to reduce the severity of insomnia. It is difficult to compare the present study’s effectiveness with that of other studies given the absence of meta-analysis studies using nonpharmacological interventions in menopausal women. Nevertheless, the results of this study are more effective for assessing the effectiveness of nonpharmacological sleep interventions in menopausal women than studies on different age groups and of women with diseases that applied nonpharmacological interventions [18,47,48]. These results suggest that nonpharmacological interventions in menopausal women, who experience sleep disorders more frequently than other sex and age groups, should be conducted more actively.

As a result of the moderating effect analysis, the variables that significantly affected sleep in menopausal women were intervention type, intervention area, and intervention duration. Acupuncture and Pilates had a significantly positive effect when the PSQI was used as an outcome variable, and in studies using the ISI as an outcome variable, meditation had a significantly positive effect. These results partially coincide with results from studies on adults in general or women of different ages [49,50]. Since the number of studies for each intervention included in the meta-analysis was small, repeated studies using each intervention are needed for a more comprehensive analysis.

When the PSQI was used as an outcome variable, the studies conducted in Asia showed a significantly larger effect size than the studies conducted in the West. These results suggest that the effect size of nonpharmacological interventions differs by culture and society due to the difference in levels of acceptance of nonpharmacological interventions and the prevalence of sleep disorders in menopausal women in different cultures and societies [51,52]. For a more thorough understanding of this phenomenon, more research is needed on the difference in the effects of nonpharmacological interventions by culture and society. In particular, among the studies included in this meta-analysis, only one study was conducted in Korea. Many experimental studies that examine the effects of nonpharmacological interventions suitable for Korean characteristics should be conducted.

With respect to intervention duration, the longer the intervention duration, the more significant the positive effect size was in the studies using the PSQI as the outcome variable. Although studies using the ISI as the outcome variable showed a positive effect, this was not statistically significant. These results are consistent with the results of previous studies [53,54] since, given the nature of nonpharmacological interventions, an intervention over a certain period of time can cause changes in the participant’s behavior. As nonpharmacological interventions have no side effects compared with pharmacological interventions, it is recommended to attempt nonpharmacological interventions before resorting to pharmacological treatments [15,55]. However, in Korea, for instance, pharmacological treatments are still commonly used to treat insomnia [56] because nonpharmacological interventions are difficult to apply in practice, although many doctors believe that nonpharmacological treatments are a highly valuable resource. In addition, the acceptability of nonpharmacological interventions may vary depending on the subjects of the nonpharmacological interventions and the methods and characteristics of the interventions; therefore, a more systematic exploration of the long-term effects of these interventions is required. Moreover, since follow-up tests were not performed in these studies except for one (33), the long-term effects of nonpharmacological interventions could not be seen. Therefore, future studies should include follow-up tests.

This study confirmed that the effect size and statistical significance of nonpharmacological interventions differed according to outcome variables such as the PSQI and ISI. These results are similar to those of a previous study that reported that the effect size of a yoga intervention was larger when measured by the PSQI than by the ISI [47]; the effect size of a moderate exercise intervention was significant when measured using the PSQI and was not significant when measured using the ISI [46]. Therefore, in a meta-analysis on the effects of nonpharmacological interventions in the future, it is necessary to separately analyze results from the PSQI, which measures sleep quality, and the ISI, which measures the severity of insomnia. In addition, since the PSQI and ISI are tools to measure the subjective quality of sleep, they can be influenced by individual emotions or feelings that can distort the results [57]. In future studies, it is necessary to objectively analyze the participants’ sleep state using objective measurement tools such as polysomnography (PSG) and actigraphy.

This study has several limitations. First, only 9 out of the 15 studies (60%) included in this study were found to have a low risk of bias in all five evaluation domains of the RoB 2.0; therefore, the effect size of this study may have been overestimated. Second, confounding factors such as the participants’ BMI, stress level, and diet may have had an effect on sleep function, but this could not be controlled. Third, the number of studies included in the review was limited since it included studies published in English and Korean only. Forth, this study used RoB 2.0 to assess risk of bias, but it is limited in sufficiently evaluating the quality of the individual studies. In future studies, it is necessary to evaluate the quality of individual studies included in the meta-analysis using the Critical Appraisal Skills Programme and Grading of Recommendations Assessment, Development, and Evaluation tools. Additionally, the small number of studies on a particular intervention makes it difficult to determine the most effective intervention.

Despite these limitations, this study is meaningful because it comprehensively reviewed the effects of nonpharmacological interventions on menopausal women with sleep disorders. These results should be interpreted carefully in accordance with the aforementioned limitations. Further research is needed to identify the effects and mechanisms of nonpharmacological interventions through high-quality studies using precise methods on a large number of people to verify the effectiveness and stability of nonpharmacological interventions in treating sleep disorders in menopausal women.

## 5. Conclusions

This meta-analysis of RCTs showed that nonpharmacological interventions significantly improved sleep quality and reduced the severity of insomnia in perimenopausal and postmenopausal women with sleep problems. Nonpharmacological interventions can be applied without any concern of side effects associated with pharmacological treatments and should be considered as a suitable intervention for sleep problems. This study is significant in that it is the first to comprehensively analyze various nonpharmacological interventions for perimenopausal and postmenopausal women with sleep problems. However, since RCTs that studied each individual intervention had a small sample size and considered less severe sleep problems, it is necessary to conduct high-quality RCTs with larger groups and more rigorous research designs to gain a better understanding of the subject.

## Figures and Tables

**Table 1:** Characteristics of included studies.

First Author (Year)	Country	Subjects		Interventions				Outcome Measure	Outcomes	Risk of Bias Summary					
		Mean Age (Year)	Sample Size (n)												
				Type of Intervention and Control Group	Length (min)	Session	Duration (Weeks)			R	D	M1	M2	S	O
Abedian (2015)	Iran	Exp1 = 50.7 Exp2 = 51.3 Cont = 51.4	Exp1 = 37 Exp2 = 36 Cont = 32	Exp1 = acupuncture Exp2 = sham acupuncture Cont = no intervention	10 10	24 24	4 4	PSQI	The total score of PSQI improved: Exp1 vs. Cont (*p* < 0.001); Exp1 vs. Exp2 (*p* < 0.001)	Ⓢ	Ⓛ	Ⓛ	Ⓛ	Ⓛ	Ⓢ
Ahmadinezhad (2017)	Iran	54.1	Exp1 = 36 Exp2 = 36 Cont = 36	Exp1 = Pilates Exp = acupuncture Cont = no intervention	60 NA	18 18	6 6	PSQI	The total score of PSQI improved: Exp1 vs. Cont (*p* < 0.001); Exp2 vs. Cont (*p* < 0.001)	Ⓛ	Ⓛ	Ⓛ	Ⓛ	Ⓛ	Ⓛ
Aibar-Almazan (2019)	Spain	Exp = 70.0 Cont = 66.8	Exp = 52 Cont = 55	Exp = Pilates Cont = no intervention	60	24	12	PSQI	The total score of PSQI improved: Exp vs. Cont (*p* < 0.001)	Ⓛ	Ⓛ	Ⓛ	Ⓛ	Ⓛ	Ⓛ
Alfonso (2012)	Brazil	Exp1 = 50.7 Exp2 = 50.7 Cont = 51.4	Exp1 = 15 Exp2 = 14 Cont = 15	Exp1 = yoga Exp2 = passive stretching Cont = no intervention	120 60	32 16	16 16	ISI	The total score of ISI improved: Exp1 vs. Cont (*p* < 0.05); Exp2 vs. Cont (*p* < 0.05)	Ⓛ	Ⓛ	Ⓛ	Ⓢ	Ⓛ	Ⓢ
Elavsky (2007)	USA	49.9	Exp1 = 63 Exp2 = 61 Cont = 39	Exp1 = walking Exp2 = yoga Cont = wait-list	60 90	48 32	16 16	PSQI	The total score of PSQI did not improve results significantly	Ⓛ	Ⓛ	Ⓛ	Ⓛ	Ⓛ	Ⓛ
Garcia (2018)	Brazil	Exp = 55.2 Cont = 56.7	Exp = 19 Cont = 11	Exp = meditation Cont = usual counseling	30	8	8	PSQI, ISI	The total score of PSQI and ISI improved: Exp vs. Cont (*p* = 0.010)	Ⓛ	Ⓛ	Ⓛ	Ⓛ	Ⓛ	Ⓛ
Green (2019)	Canada	Exp = 53.3 Cont = 52.9	Exp = 37 Cont = 34	Exp = CBT Cont = wait-list	120	12	12	PSQI	The total score of PSQI improved: Exp vs. Cont (*p* = 0.001)	Ⓛ	Ⓛ	Ⓛ	Ⓛ	Ⓛ	Ⓛ
Hachul (2013)	Brazil	Exp = 58.0 Cont = 59.8	Exp = 9 Cont = 9	Exp = acupuncture Cont = sham acupuncture	NA	10	5	PSQI	The total score of PSQI improved: Exp1 vs. Cont (*p* < 0.001)	Ⓗ	Ⓛ	Ⓛ	Ⓛ	Ⓛ	Ⓗ
Ham (2020)	South Korea	Exp = 53.8 Cont = 55.5	Exp = 28 Cont = 30	Exp = CBT Cont = usual education	30–60	5	4	PSQI, ISI	The total score of PSQI and ISI improved: Exp vs. Cont (*p* < 0.05)	Ⓛ	Ⓛ	Ⓛ	Ⓛ	Ⓛ	Ⓛ
Lee (2020)	China	Exp = 52.1 Cont = 53.1	Exp = 42 Cont = 42	Exp = acupuncture Cont = sham acupuncture	NA	18	8	PSQI	The total score of PSQI improved: Exp1 vs. Cont (*p* < 0.001)	Ⓛ	Ⓛ	Ⓛ	Ⓛ	Ⓛ	Ⓛ
Lucena (2021)	Brazil	Exp = 56.7 Cont = 55.9	Exp = 17 Cont = 18	Exp = aromatherapy Cont = placebo	NA	28	4	PSQI, ISI	The total score of PSQI and ISI improved: no statistically significant findings; Exp vs. Cont (*p* = 0.220)	Ⓛ	Ⓛ	Ⓛ	Ⓛ	Ⓛ	Ⓛ
McCurry (2016)	USA	Exp = 55.0 Cont = 54.7	Exp = 53 Cont = 53	Exp = CBT Cont = usual education	20–30	6	8	PSQI, ISI	The total score of PSQI and ISI improved: Exp vs. Cont (*p* < 0.001)	Ⓛ	Ⓛ	Ⓛ	Ⓛ	Ⓛ	Ⓛ
Portella (2021)	Brazil	Exp = 46.7 Cont = 48.6	Exp = 18 Cont = 15	Exp = meditation + sleep hygiene Cont = sleep hygiene	45	56	8	PSQI, ISI	The total score of PSQI and ISI did not improve: Exp vs. Cont (*p* = 0.492) in PSQI Exp vs. Cont (*p* = 0.278) in ISI	Ⓢ	Ⓛ	Ⓛ	Ⓢ	Ⓛ	Ⓢ
Pu (2017)	China	Exp = 52.0 Cont = 52.5	Exp = 37 Cont = 37	Exp = acupuncture Cont = sham acupuncture	NA	10	3	PSQI, ISI	The total score of PSQI and ISI improved: Exp vs. Cont (*p* < 0.01)	Ⓛ	Ⓛ	Ⓗ	Ⓛ	Ⓢ	Ⓗ
Tadayon (2016)	Iran	Exp = 52.3 Cont = 52.5	Exp = 56 Cont = 56	Exp = walking Cont = no intervention	NA	NA	12	PSQI	The total score of PSQI improved: Exp1 vs. Cont (*p* = 0.001)	Ⓗ	Ⓛ	Ⓛ	Ⓗ	Ⓢ	Ⓗ

Exp—experimental group; Cont—control group; ISI—Insomnia Severity Index; PSQ—Pittsburg Sleep Quality Index; R—randomization process; D—deviations from intended interventions; M1—missing outcome data; M2—measurement of the outcome; S—selection of the reported result; O—overall bias; Ⓛ—low risk of bias; Ⓗ—high risk of bias; Ⓢ—some concern.

**Table 2:** Modulator Analyses of Nonpharmacological Intervention in Perimenopausal Women with Sleep Problems.

Categorical Modulators	PSQI				ISI			
	N	ES	95% CI	*p*	N	ES	95% CI	*p*
Type of intervention								
Acupuncture	5	−2.11	−3.04, −1.17	0.392	1	−1.81	−6.18, 2.55	0.624
Pilates	2	−1.78	−3.22, −0.33					
Exercise	2	−0.90	−2.32, 0.52					
Yoga	1	−0.11	−2.11, 1.90		1	−0.61	−4.99, 3.79	
Meditation	2	−3.24	−2.86, 0.21		2	−2.45	−5.62, −0.72	
CBT	3	−0.61	−1.78, 0.56		2	−0.66	−3.74, 2.43	
Aromatherapy	1	−1.16	−3.26, 0.93		1	−0.65	−5.04, 3.73	
Study region								
Asia	7	−1.94	−2.59, −1.30	0.010 *	2	−1.12	−3.28, 1.05	0.866
Western	9	−0.81	−1.39, −0.23		5	−1.34	−2.74, 0.06	
Control type								
No intervention	8	−1.36	−2.11, −0.61	0.664	1	−0.61	−4.09, 2.88	0.906
Other	4	−0.96	−2.05, 0.14		4	−1.49	−3.25, 0.27	
Placebo	4	−1.67	−2.77, −0.57		2	−1.24	−3.69, 1.21	
Continuous modulators								
Sample size	16		−0.01, 0.06	0.169	7		−0.09, 0.10	0.910
Duration of intervention	16		0.03, 0.25	0.010 *	7		−0.27, 0.30	0.903
Total length of class	9		0.00, 0.01	0.019 *	5		−0.01, 0.00	0.441

CBT—cognitive behavioral therapy; CI—confidence interval; ES—effect size (Hedges’ g); ISI—Insomnia Severity Index; PSQI—Pittsburgh Sleep Quality Index; * *p* < 0.05 indicates a significant difference.

## Data Availability

Not applicable.
